# School-based Intervention to Promote Healthy Lifestyles in Sousse, Tunisia

**DOI:** 10.4103/0970-0218.62581

**Published:** 2010-01

**Authors:** Imed Harrabi, Jihene Maatoug, Mehdi Gaha, Raoudha Kebaili, Rafika Gaha, Hassen Ghannem

**Affiliations:** Service of Epidemiology and Biostatistics, University Hospital Farhat Hached - 4000 Sousse, Tunisia

**Keywords:** Child and adolescent health, community health, risk behaviours

## Abstract

**Background:**

Integrated actions against selected risk factors (i.e. smoking, physical inactivity, and unhealthy diet) can lead to the reduction of major chronic diseases.

**Objective:**

To implement and evaluate a school-based intervention program to prevent cardiovascular risk factors among children.

**Materials and Methods:**

**Design:**

Pre- test post-test quasi experimental design with a control group.

**Setting:**

Four secondary schools in Sousse, Tunisia.

**Intervention:**

The overall intervention program lasted for a school year and incorporated educative actions concerning tobacco use, physical activity, and healthy diet.

**Results:**

Globally, knowledge, behaviors, and intentions concerning smoking improved in both groups between baseline and the end of the study, particularly in the intervention group. Nutrition knowledge, behaviors, and intentions improved in both groups between baseline and final stage, particularly in the intervention group. At the final stage, there was an increase in the proportion of children walking to and from school in the intervention group. There was also an increase in the percentage of children with intention of practicing sport in the future particularly in the intervention group. There were no significant differences in BMI after the intervention neither in intervention nor in control groups. At the end of the study, the incidence of overweight and obesity was similar to that at baseline.

**Conclusions:**

This pilot study has demonstrated the potential of school as a suitable setting for the promotion of healthy lifestyles in children. The study resulted in substantial improvements concerning knowledge, behaviors, and intentions in the intervention group.

## Introduction

Chronic diseases and particularly cardiovascular diseases (CVD) are still the major causes of deaths in most developed countries,(1) despite the downward trends observed during the last three decades.(2) Their risk factors are also well known in most industrialized countries,(3,4) where it has been proceeded to the implementation of effective preventive programs.(5)

Although CVD are emerging in developing countries, little is known about comprehensive preventive measures for controlling their expansion.(6)

Tunisia is also concerned and is now facing the epidemiological transition phenomenon(7) with a decrease in total mortality, increase of life expectancy and adoption of lifestyles associated with non-communicable diseases, diabetes, and CVD in particular.(8)

Integrated actions against selected risk factors (i.e. smoking, physical inactivity and unhealthy diet) can lead to the reduction of major chronic diseases and particularly CVD. These interventions should take place early in childhood. In fact, the need for early intervention to promote cardiovascular health in children is recognized(9) because children exhibit risk factors for CVD that often persist into adulthood. Many researchers recommend a population approach to improve cardiovascular health behaviors in children.(10,11) This can be done through teaching and other interventions provided to all children in a variety of settings such as schools and other community locations.

Many school-based interventions to promote healthy lifestyles were implemented all over the world. However, currently, there are limited quality data on the effectiveness of such programs. Thus, generalizable conclusions cannot be drawn.

At the inception of this study, there had been no school-based preventive work undertaken in Tunisia and even in North Africa, which made it an important pilot study.

The objective of the present study was to evaluate the effect of a school cardiovascular disease risk factors prevention program on the knowledge and intentions of pupils aged 12-16 in Sousse, Tunisia.

## Materials and Methods

### Design

This study adopted a pre-post quasi-experimental design, and divided the subjects into two groups. The intervention group was subject to classroom-based cardiovascular disease risk factors prevention curriculum, while the control group received no intervention. One month before the intervention began, and, separately, a month after it ended, the intervention group and the control group were tested, so that effects of the intervention could be ascertained.

### Population

The study concerned pupils of secondary public schools in Sousse, Tunisia, aged 12-16. Two districts from the city of Sousse (Sousse Jawhara, Sousse Riadh) served, respectively, for the intervention and control groups. The intervention was implemented in two public schools: Ezzahra and Khzema Ouest and concerned the totality of the pupils (total number = 1965). Two control public schools were selected: Ezzouhour and Essalem (total number = 1737). The selection of schools was based on age, socioeconomic, and demographic characteristics. It was also based on the students' number in each school.

The pre- and post-evaluations were applied to a representative sample in each secondary school, which was based on these parameters:

α = 5%;

β= 20% so the power of the study was 80%.

An increase of 10% in the knowledge of the schoolchildren after the intervention study about the healthy nutrition, the benefit of physical activity, and the consequences of smoking habit.

The calculated minimal sample size was 958 students in each group. We majored this number by 10%, considering the possible dropped out, so we obtained 1054 students in each group, i.e. 30 classes of 35 students in each group. As some classes contained less than 35 students, we included more than 30 classes per group (intervention: 39, control: 37) through a stratified and proportional sampling in each secondary school.

Thus, the final number of students who participated to the first evaluation was 2338.

**Figure 1:**
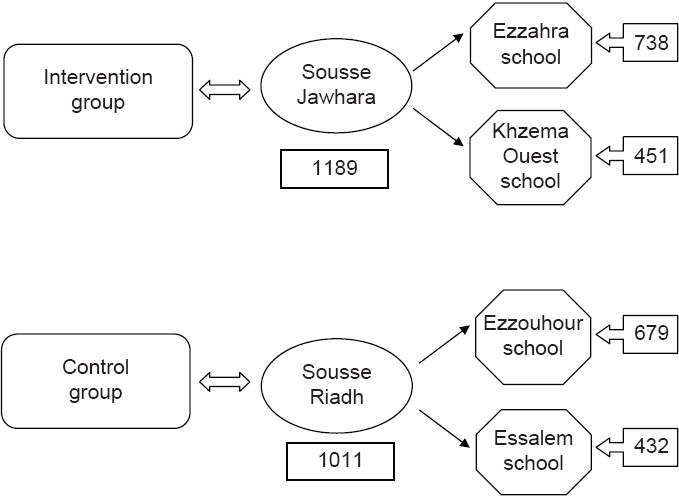
School-based intervention sample to promote healthy lifestyles in Sousse, Tunisia, 2007

At the second evaluation at the end of the year, 138 students were dropped out (intervention: 58, control: 80). They either changed from one class to another, one college to another, or were absent from class during the final evaluation. So, 2200 students participated effectively to the pre- and post-evaluations [Figure 1].

### Intervention

The overall intervention program lasted for a school year and incorporated educative actions concerning the main CVD risk factors: Tobacco use, physical activity, and healthy diet.

Interventions were delivered by team of the project with the collaboration of teachers and school doctors. The school-based intervention program integrated biological sciences and physical education that were part of the high school's curriculum.

The first step was a 60 min theoretical session concerning one of the three items (tobacco prevention, healthy diet, and regular physical activity), which provided the cognitive behavioral components of health knowledge and health promoting concepts.

During the following 4 weeks, the students were asked to prepare a production such as a poster, a piece of theatre, a poem, or any other production concerning the item in question, so that they could effectuate self-research and get more impregnated with the subject.

The third step took place 4 weeks later, when students presented their productions in class during a 60-min session, and discussed them with their classmates and responsible teacher.

**Figure 2:**
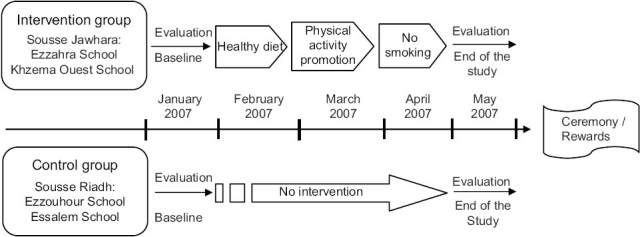
School-based intervention design to promote healthy lifestyles in Sousse, Tunisia, 2007

These steps were repeated twice so that the three items were presented and discussed during 3 months [Figure 2].

A brief outline of the program is given below:

### Smoking intervention program

- Present epidemiological data concerning smoking in Tunisia and over the world.
- Present the main components of a cigarette and their effects on health.
- Discuss incentives of pupils who begin smoking.
- Explain consequences of tobacco use: Psychological, physical, economical, and social effects.
- Explain the phenomenon and the consequences of second hand smoking.

### Nutrition intervention program

- Explain principles of dietary pyramid.
- Discuss the ideal composition of principal meals.
- Give examples of healthy and unhealthy foods and present their benefits/misdeeds.
- Explain the importance of breakfast and its place in dietary balance.

### Physical activity intervention program

- Explain benefits of non-competitive physical activity.
- Explain how to practice physical activity (which sport, how long, how much …).
- Promote activity in daily life.
- Encourage all pupils' even girls and obese children to participate.

During the whole intervention, interclass sport tournaments were organized.

Students who were part of health clubs, generally 2-3 h a week, animated by biological science teacher-, could discuss more about CVD risk factors and healthy lifestyles.

At the end of the school year, a ceremony was given with representatives of public health ministry, education ministry, celebrities, members of the team who participated to the implementation of the program, and all pupils of intervention group with their families. The best productions of students were presented, quiz-games concerning healthy lifestyles were organized, and winners were rewarded.

### Variables and their measurement

We used a pre-tested self-administered questionnaire. Data collected by the questionnaire concerned: Socio-demographic variables (age, sex, parents' education,…); variables concerning pupils' knowledge, behaviors, and intentions about smoking, dietary habits, and physical activity.

Anthropometric data: Height and weight were carried out with the children wearing only underclothes and no shoes. Body weight was recorded to the nearest 0.1 kg using a standard beam balance scale. Body height was recorded to the nearest 0.5 cm.

Body mass index (BMI) was computed, as the ratio of the body weight to the body height squared expressed as kg/m^2^. To define overweight and obesity, we used the recent international cut-off values of BMI.(12)

### Statistical analysis

Statistical analysis was performed using the Statistical Package for Social Sciences (SPSS 10.0). Data are presented as frequencies, means, and standard deviations. The *t*-test and Chi-square test were used, respectively, to compare means and percentages in and between groups. Statistical significance was set at *P* < 0.05.

### Ethnical considerations

Because of the young age of the target population, this investigation was undertaken with prudence, and with respect of the rights and the integrity of people. Parents gave their consent and they were able to refuse their children participation. We used an anonymous questionnaire that did not contain the name or the address of students.

## Results

### Intervention group

It included 1189 children, 556 (46.8%) boys and 633 (53.2) girls, aged 12-16 (mean ± SD = 13.3 ± 1.1 years).

Father's education level was primary school in 22.7%, secondary school in 39%, and higher education in 26.9%. Only 1.2% were illiterate and 10.3% did not know their father's education level.

Percentages concerning mother's education level were respectively 30.3%, 35.3%, 19.2%, 6.2%, and 9%.

For parents' profession, 1.6% of fathers and 62.9% of mothers were unemployed.

### Control group

It included 1011 children, 470 (46.5%) boys and 541 (53.5) girls, aged 12-16 (mean ± SD = 13.5 ± 1.2 years).

Father's education level was primary school in 34.4%, secondary school in 40.6%, and higher education in 20.4%. Only 4.4% were illiterate and 0.3% did not know their father's education level.

Percentages concerning mother's education level were respectively 44.3%, 27.6%, 10.9%, 17.1%, and 0.1%.

For parents' profession, 1.6% of fathers and 76.9% of mothers were unemployed.

### Tobacco use

Knowledge of tobacco health effects, in terms of percentages of students who answered right to the question ‘Does smoking cause CVD?’, was improved from pre- to post-questionnaire, in both control and intervention groups. But it was significant in the last one [Table 1].

**Table 1:** Comparison of knowledge, behaviors, and intentions concerning smoking in intervention and control groups after 1 year follow-up, Sousse, Tunisia, 2007

	Baseline	After the intervention	Degree of significance *P* value*
Smoking causes CVD			
Intervention group (*n* = 1189)	11.5	46.3	<10^−3^
Control group (*n* = 1011)	14.1	14.6	0.74
Degree of significance			
*P* value**	0.06	<10^−3^	
Smoking causes lung neoplasm			
Intervention group (*n* = 1189)	49.1	84.9	<10^−3^
Control group (*n* = 1011)	61.3	68.6	<10^−3^
Degree of significance			
*P* value**	0.98	<10^−3^	
Smokers			
Intervention group (*n* = 1189)	4.7	3.2	<10^−3^
Control group (*n* = 1011)	7.6	5.9	<10^−3^
Degree of significance			
*P* value**	0.004	0.002	

* Comparison between pre- and post-evaluations for each group (ex: In intervention group, 11.5% answered right to the question “Does smoking cause CVD?” at baseline, and 46.3% answered it right at the post evaluation)

** Comparison between the intervention group and control group at pre- and post-evaluation

The degree of change from baseline in percentage of knowledge that smoking causes lung neoplasm was significantly different between intervention and control groups (+35.8% vs. +7.3%; *P* < 10^−3^)

The intention to smoke in the future decreased significantly in the intervention group but increased in the control one [Table 1].

The smoking behavior decreased significantly in both groups and the percentage of change was not statistically different between the two groups (−1.5% in the intervention group vs. 1.8% in the control group; *P* = 0.62) [Table 1].

### Nutrition knowledge, behaviors, and intentions

The percentage of students who know what should they eat on breakfast have been improved significantly in the intervention group (15.4-40.5%; *P*<10^−3^) but not in the control one (16.4-17.6; p=0.28) [Table 2].

**Table 2:** Comparison of knowledge, behaviors, and intentions concerning nutrition in intervention and control groups after 1 year follow-up, Sousse, Tunisia, 2007

	Baseline	After the intervention	Degree of *P* value*
Do you know what should you eat on breakfast?			
Intervention group (*n* = 1189)	15.4	40.5	<10^−3^
Control group (*n* = 1011)	16.4	17.6	0.28
Degree of significance			
*P* value**	0.53	<10^−3^	
Will you eat breakfast in the future?			
Intervention group (*n* = 1189)	83.6	91.8	<10^−3^
Control group (*n* = 1011)	81.6	84.5	0.002
Degree of significance			
*P* value**	0.22	<10^−3^	
Do you eat at least 5 fruits and vegetables per day?			
Intervention group (*n* = 1189)	45.3	55.4	0.06
Control group (*n* = 1011)	48.3	57.9	0.03
Degree of significance			
*P* value**	0.42	0.49	

* Comparison between pre- and post-evaluation for each group.

** Comparison between the intervention group and control group at pre- and post-evaluation

The intention to eat breakfast in the future has been improved in the two groups [Table 2]. But the percentage of change was significantly higher in the intervention group (+8.2% vs. +2.9%; *P* < 10^−3^).

Students who eat more than five fruits and vegetables per day increased in the two groups but this improvement was not significant in the intervention group [Table 2].

### Physical activity

The intention to practice physical activity in the future increased in the intervention group (87.1- 96.2%; *P* < 10^−3^), but not in the control group (91-92.7%; *P* = 0.15) [Table 3]. After the intervention, students who practice more than 30 min of physical activity for at least 6 days a week increased significantly in the two groups but the percentage of change was significantly higher in the intervention group (+18.4% vs. +9.7%; *P* < 10^−3^) [Table 3].

**Table 3:** Comparison of knowledge and intentions concerning physical activity in intervention and control groups after one year follow-up, Sousse, Tunisia, 2007

	Baseline	After the intervention	Degree of significance *P* value*
Intention to practice physical activity daily			
Intervention group (*n* = 1189)	87.1	96.2	<10−^3^
Control group (*n* = 1011)	91.0	92.7	0.15
Degree of significance			
*P* value**	0.004		
Practice more than 30 mn of physical activity for at least six days a week*			
Intervention group (n= 1189)	17.5	35.9	<10^−3^
Control group (*n* = 1011)	27.2	36.9	<10^−3^
Degree of significance			
*P* value**	<10^−3^	<10^−3^	

* Comparison between pre and post evaluation for each group.

** Comparison between the intervention group and control group at pre- and post-evaluation

## Discussion

The purpose of a pilot study includes the assessment of feasibility, efficacy, and acceptability which in turn will lead to a decision on whether or not to pursue a particular approach. This is a valuable pilot study for Tunisia and North Africa that has used the school setting in an innovative way to promote healthy lifestyles and to prevent cardiovascular risk factors.

After 1 year of intervention, the program was found to be acceptable to school administrators, teachers, parents, and children.

The findings of this study suggest that school-based intervention can change lifestyle knowledge and intention in a short period, but the behavior effect would be more difficult to change particularly in smoking behavior. Small net changes in the favorable direction were observed for physical activity and smoking. So, intervention programs in schools may, after sufficient duration, prove to be effective in reducing the population risk for the future development of chronic disease.(13) In fact, the primary prevention of chronic disease should begin in childhood, but the effect of such programs on knowledge precedes behaviors.(14) Kreuter and *et al.*(15) think that attempts to establish immediate behavior change as goals of school programs would be naive. Federal and state initiatives are needed to support the necessary cost-benefit studies and the long-term evaluations required for policy decisions concerning school health education programs. Local support is adequate to perform short-term studies of impact on knowledge and skills of specific health education programs.(15)

In the San Diego Family Health Project for example(16) diet changed somewhat, but activity changed only minimally.

An improvement concerning knowledge, behaviors, and intentions was also seen in the control group but was less than in the intervention one. Other studies with significant effects have involved parents in the intervention.(17) We chose to test a simpler and more practical model and thus limited the interventions to the school day and to children only.

A true experimental design would have a number of communities allocated randomly into intervention and control ones. This is seldom possible, as it may not comply with the basic idea of comprehensive community interventions. Instead, quasi-experimental designs have been often used with a reference community or with the national change as the comparator.(18)

If these findings can be replicated, the widespread implementation of such programs has the potential to reduce the population risk for the future development of the nation's leading causes of premature mortality.(19)

An evaluative study concluded that school-based health education programs have three important roles in community health promotion. First, the provision of a fundamental understanding of health and disease concepts to large segments of the population. Secondly, the reinforcement of positive health attitudes, and thirdly, the alteration of concurrent health behaviors for significant health problems.(20)

## Conclusion

The study resulted in substantial improvements concerning knowledge, behaviors, and intentions in the intervention group. Because there is evidence that risk factors continue into adulthood, it is logical to assume that an intervention that reduces mean levels of risk factors in a majority of the children, if carried out for a longer period of time, could eventually have an impact on mortality rates and morbidity. A more elaborate level of action consists in realizing an integrated community-based program of non-communicable disease prevention. This program will target the young as well as the adults by the implementation of various educational activities with respect to lifestyles in the general frame of a community mobilization perspective.
